# Predictors of Frequent Emergency Room Visits among a Homeless Population

**DOI:** 10.1371/journal.pone.0124552

**Published:** 2015-04-23

**Authors:** Kinna Thakarar, Jake R. Morgan, Jessie M. Gaeta, Carole Hohl, Mari-Lynn Drainoni

**Affiliations:** 1 Section of Infectious Diseases, Boston Medical Center, Boston, Massachusetts; 2 Department of Health Policy and Management, Boston University School of Public Health, Boston, Massachusetts; 3 Boston Health Care for the Homeless Program, Boston, Massachusetts; 4 Section of General Internal Medicine, Boston Medical Center, Boston, Massachusetts; 5 Center for Healthcare Organization and Implementation Research, ENRM Memorial VA Hospital, Bedford, Massachusetts; Institute of Psychiatry, UNITED KINGDOM

## Abstract

**Background:**

Homelessness, HIV, and substance use are interwoven problems. Furthermore, homeless individuals are frequent users of emergency services. The main purpose of this study was to identify risk factors for frequent emergency room (ER) visits and to examine the effects of housing status and HIV serostatus on ER utilization. The second purpose was to identify risk factors for frequent ER visits in patients with a history of illicit drug use.

**Methods:**

A retrospective analysis was performed on 412 patients enrolled in a Boston-based health care for the homeless program (HCH). This study population was selected as a 2:1 HIV seronegative versus HIV seropositive match based on age, sex, and housing status. A subgroup analysis was performed on 287 patients with history of illicit drug use. Chart data were analyzed to compare demographics, health characteristics, and health service utilization. Results were stratified by housing status. Logistic models using generalized estimating equations were used to predict frequent ER visits.

**Results:**

In homeless patients, hepatitis C was the only predictor of frequent ER visits (OR 4.49, p<0.01). HIV seropositivity was not predictive of frequent ER visits. In patients with history of illicit drug use, mental health (OR 2.53, 95% CI 1.07–5.95) and hepatitis C (OR 2.85, 95% CI 1.37–5.93) were predictors of frequent ER use. HIV seropositivity did not predict ER use (OR 0.45, 95% CI 0.21 – 0.97).

**Conclusions:**

In a HCH population, hepatitis C predicted frequent ER visits in homeless patients. HIV seropositivity did not predict frequent ER visits, likely because HIV seropositive HCH patients are engaged in care. In patients with history of illicit drug use, hepatitis C and mental health disorders predicted frequent ER visits. Supportive housing for patients with mental health disorders and hepatitis C may help prevent unnecessary ER visits in this population.

## Introduction

According to the U.S. Department of Housing and Urban Development, approximately 1.59 million individuals spent at least one night in shelter in 2010 [1]. Homeless individuals experience high disease burdens and mortality rates [2–4]. Studies have shown that homeless individuals are frequent emergency room (ER) users and have high health care expenditures [5–6]. In a recent analysis of costs of 6,494 persons served by a Boston HCH program, total annual expenditures due to ER visits was estimated as $16,011,738 annually. In the same study, it was found that approximately half of the total expenditures were incurred by just 10% of the study population [6]. Multiple factors have been identified as predictors of frequent ER use in homeless persons such as older age, previous hospital admissions and emergency room visits, multiple primary care visits, perceived inadequate mental health care, poor health status, and HIV [7–9]. Several clinical trials have shown that interventions such as intensive case management programs [10–11], assertive community treatment teams [12–13], or supportive housing [14–18] can reduce frequent ER visits and hospital costs.

Substance use has a particularly complex relationship with homelessness, as it can contribute to, and be worsened by, homelessness. In a study involving over 28,000 people experiencing homelessness in Boston from 2003–2008, drug overdose was found to be the leading cause of death among this population [19]. In the same study, mortality rates in homeless men and women were compared to the state population; it was found that mortality due to drug overdose was 16 to 24 times higher in homeless persons compared with age and sex-matched controls in the general population [19]. Additionally, substance use disorders have been shown to independently increase risk for first-time homelessness, and unstable housing has been associated with higher levels of drug use [20–21]. For example, individuals with unstable housing report nearly twice as much receptive sharing of needles, or injecting a needle used by another person, compared to patients with stable housing [21].

The first purpose of this study was to identify risk factors for frequent ER visits in a sample of Health Care for the Homeless Program (HCH) patients. Specifically, the effect of housing status on risk factors for frequent ER visits was examined. Differences in demographics, comorbidities and health service utilization in homeless versus non-homeless individuals have been cited as important predictors of emergency room utilization [22–23], but few studies have explicitly examined the differences in magnitude of the predictors between homeless and non-homeless populations. We believed that certain health characteristics, particularly involving substance use, would be associated with frequent ER use in homeless individuals, as compared to the housed individuals, given the prevalence of drug overdose in the homeless population [19]. Since housing instability has been associated with ER use [2], we also hypothesized that homeless individuals engaging in ambulatory care would have a higher magnitude of frequent ER visits compared to housed individuals also accessing ambulatory care. Therefore, the results of this study were stratified in order to capture differences in predictor variables by housing status. The second purpose of the study was to identify risk factors for ER visits specifically in those HCH patients with a history of illicit drug use. This study population is unique in that participants have access to comprehensive outpatient services (primary care, primary HIV care, behavioral health, medical respite, and case management). The results of this study will help identify characteristics of individuals who are frequent ER users in order to ascertain areas for targeting services to reduce ER visits in this population.

## Methods

### Study setting

This study was a retrospective analysis of a convenience sample of 412 adult patients enrolled in a HCH in Boston. The HCH delivers comprehensive services, including outreach on the streets, primary care, primary HIV care, case management, behavioral health care, and medical respite care. The program currently serves over 12, 511 individuals annually with more than 70 service sites in the Boston area. The only criterion for enrollment is that participants must be homeless at the time of enrollment.

### Data Sources

Data for the study came from the electronic medical records (EMR) of both the HCH and an urban safety net medical center located directly across the street from the HCH’s largest clinic. Although the HCH participants are not exclusively admitted to the safety net hospital, it does serve as a main site of ER care for many HCH patients. The study population were initially selected as a 2:1 HIV seronegative versus HIV seropositive match based on age, sex, and housing status for a separate analysis examining differences in disease burden and health care utilization in HIV seronegative versus seropositive individuals. The number of HIV seropositive patients seen regularly in the HCH program was known (n = 187). Given the small sample of HIV seropositive participants, a 2:1 match of HIV seronegative to seropositive patients was performed in order to make the sample size more robust for analyzing multiple outcomes. In addition to age and sex, housing status was included as a matched variable because, based on prior studies, it was thought that homelessness would have a powerful effect on outcomes.

Data from July 1, 2011 – June 30, 2013 were extracted from the EMRs from both the HCH and the safety net hospital and provided as a single dataset to the research team. Data extracted from the HCH EMR included demographics, health characteristics (including substance use, HIV, hepatitis C, and other chronic conditions), and ambulatory (primary care, respite stays, behavioral health, case management) visits. Data extracted from the safety net hospital EMR included ER visits. All patients selected for the study had an index ambulatory care visit between July 1, 2011 and June 30, 2012. Health service utilization data were restricted to a one-year time period following the index ambulatory care visit, in order to ensure equal follow-up time for all study patients.

### Inclusion criteria

Patients included in the study were age 18 years or older, enrolled in the HCH and had at least one ambulatory care visit to HCH between July 1, 2011 and June 20, 2012.

### Outcome variable

The primary outcome of the study was frequent ER visits. For this study, “frequent” ER visits were defined as two or more ER visits to the medical center within one-year following the index HCH ambulatory care visit.

### Housing Status

Patients were categorized as housed versus homeless at the time of the index ambulatory visit. “Housed” was defined as any patient whose housing status was listed in the EMR as supportive housing, housing without supportive services, assisted living, nursing home, or rest home. “Homeless” was defined as any patient living in a shelter, transitional housing, doubled up, living on the street, or whose housing status was “other” or “unknown.”

### Covariates

Covariates included demographics, health characteristics and ambulatory visits. The definitions for these variables are provided below.

### Housing changes

In order to account for patients who experience episodic homelessness, a categorical variable was created to capture housing stability using the frequency of housing changes. “No housing change” was defined as no change in housing status (“homeless” to “housed,” or vice versa) within the one year review period. “Any housing change” was defined as one or more changes in housing status during the review period.

### Health characteristics

Prognostic comorbidity was calculated using the Charlson comorbidity index, which utilizes a weighted score of seventeen comorbidities to help predict long term mortality [24]. All health characteristics for the comorbidity index, such as diabetes, renal disease, chronic pulmonary disease, and malignancy, were defined using International Classification of Diseases, Ninth Revision (ICD-9) coding. If any of these problems appeared on the patient's problem list in the EMR, they were also included in the analysis. For purposes of this study, where examining the effects of HIV seropositivity on the outcome was of particular interest, HIV serostatus was excluded from the comorbidity index. A high Charlson comorbidity index score was defined as any score of greater than one (excluding HIV).

In terms of other health characteristic variables, *mental health disorder* was based on any ICD-9 codes for a mental health condition. For both hepatitis C and HIV, in addition to relying on ICD-9 codes, laboratory data (reactive HIV antigen and/or antibodies, detectable HIV viral loads, reactive hepatitis C antibodies plus detectable hepatitis C viral loads) were used to more accurately capture these diagnoses in patients. *Tobacco use* was defined using ICD-9 codes or patient self-report of tobacco use. *Alcohol use* was defined using ICD-9 codes and patient self-report, or of attendance at Alcoholics Anonymous. *Illicit drug use* was also defined using ICD-9 codes, patient self-report, or positive toxicology screens for cocaine, methamphetamines, opioids or benzodiazepines (if opioids or benzodiazepines were not on the patient's medication list). *Albumin level* was determined using laboratory data from the HCH clinical database, with a cutoff of > 3.5 g/dL defined as high albumin level.

### Ambulatory visits

Ambulatory visits included primary care, medical respite stays, behavioral health, and case management visits. All ambulatory visits were divided into categorical variables: 0–2 visits versus 3 or more visits during the one-year follow-up period.

*Primary care visits* were defined as outpatient primary care visits with physicians, nurse practitioners, physician assistants, or nurses, including those encounters that occurred in outreach settings such as shelters or street. *Respite stays* were defined as any patient who had stays at the HCH medical respite facility. *Behavioral health visits* were defined as any pharmacologic mental health visits or group/individual mental health or substance abuse treatment visits. *Case management visits* included any visits with a HCH case manager.

### Statistical Analysis

Descriptive and multivariable analyses were performed in this study. Chi-square statistics were used to examine differences in proportions when analyzing demographics (age, sex, race), health characteristics (Charlson comorbidity index scores, mental health disorder, hepatitis C, HIV, history of tobacco, alcohol or illicit drug use,), and health care utilization (primary care visits, mental health care visits, case management visits, medical respite stays) by housing status.

Univariate and multivariable logistic regression analyses were performed to analyze factors associated with frequent ER use. The main results were stratified by housing status to explicitly examine the differences in predictors of ER use for homeless and housed populations. This multivariable analysis took into account clustering by matched study participants using generalized estimating equations with an exchangeable correlation structure (Table 2). Another multivariable analysis was performed using logistic regression of pooled (i.e. unstratified) data without taking into account clustering of matched study participants (Table 3). All covariates were used in the multiviariable analyses as the models were based on clinical relevance of variables, and there was no significant collinearity between variables. All reported p-values were two-sided; an alpha level of 0.05 was considered to indicate statistical significance. A subgroup analysis was performed on 287 patients with history of illicit drug use to address the second study objective.

STATA version 13.1 (College Station, TX) was used for the analysis. This study was approved by the Boston University Medical Campus Institutional Review Board (BUMC IRB). Because the investigators did not have access to protected health information, it was not possible to obtain consent from study participants since data were anonymous. The study qualified as exempt, and a Health Insurance Portability and Accountability Act waiver of authorization was approved by the BUMC IRB.

## Results

### Description of Homeless Patients vs. Housed Patients

**Table 1** describes the overall study population, comparing the homeless and housed patients. Housed patients were older (mean age 52.5 +/- 5.9 years) compared to homeless patients (mean age 49.1 +/- 6.9 years, p = 0.002). Housed individuals were more likely to have housing stability (94% vs. 86%, p = 0.01), history of alcohol use (82% vs. 71%, p = 0.01), mental health disorder (74% vs. 62%, p = 0.02), and have higher albumin levels (61 vs. 36%, p<0.001). There were no statistically significant differences in race, Charlson comorbidity index scores, hepatitis C, tobacco use, or illicit drug use by housing status. There was also no difference based on HIV serostatus **(Table 1)**. When comparing ambulatory service use by housing status, housed patients were more likely to have three or more primary care visits (89% vs. 76%, p = 0.002) and case management visits (34% vs. 18%, p<0.001). There were no significant differences in frequent ER visits (13% versus 15% in housed patients and homeless patients, respectively, p = 0.59) behavioral health visits (4% vs. 3%, p = 0.80), or respite care stays (16% vs. 24%, p = 0.06), by housing status in the descriptive analysis **(Table 1)**.

**Table 1:** Baseline Characteristics of Study Population by Housing Status.

	Total	Housed patients	Homeless patients	p value
	n = 412	n = 134	n = 278	
	n(%)	n(%)	n(%)	
**Demographics**				
Mean age in years (SD)	50.2 (6.8)	52.5 +/- 5.9	49.1 (6.9)	
**Age in years** ^**a**^				
18–54	291 (71)	81 (60)	210 (76)	
≥ 55	121 (29)	53 (40)	68 (24)	0.002
**Sex**				
Male	315 (76)	101 (75)	214 (77)	
Female	97 (24)	33 (25)	64 (23)	0.72
**Race**				
Minority/Unreported	298 (70)	94 (70)	195 (70)	
White	123 (30)	40 (30)	83 (30)	0.99
**Housing changes** ^**a**^ ^**b**^				
No housing changes	364 (88)	126 (94)	238 (86)	
1+ housing change	48 (12)	8 (6)	40 (14)	0.01
**Health characteristics**				
Illicit Drug use	287 (70)	92 (69)	195 (70)	0.76
Alcohol use^**a**^	307 (75)	110 (82)	197 (71)	0.01
Tobacco use	237 (58)	84 (63)	153 (55)	0.14
HIV seropositive	138 (34)	47 (35)	91 (33)	0.64
Mental Health disorder^**a**^	272 (66)	99 (74)	173 (62)	0.02
Hepatitis C	137 (34)	42 (31)	95 (34)	0.57
Charlson Index >1(excluding HIV)	31 (8)	14 (10)	17 (6)	0.12
**Albumin** ^**a**^				
0–3.5 g/dL	21 (5)	10 (8)	11 (4)	
> 3.5 g/dL	182 (44)	82 (61)	100 (36)	
Missing	209 (51)	42 (31)	167 (60)	<0.001
**Utilization** ^**c**^				
**Emergency room**				
0–1 visits	351 (86)	116 (87)	235 (85)	
2+ visits	61 (14)	18 (13)	43 (15)	0.59
**Primary care** ^**a**^				
0–2 visits	82 (20)	15 (11)	67 (24)	
3+ visits	330 (80)	119 (89)	211 (76)	0.002
**Behavioral health**				
0–2 visits	398 (79)	129 (96)	269 (97)	
3+ visits	14 (3)	5 (4)	9 (3)	0.80
**Respite stays** ^**a**^				
0–2 visits	325 (79)	113 (84)	212 (76)	
3+ visits	87 (21)	21 (16)	66 (24)	0.06
**Case management** ^**a**^				
0–2 visits	317 (77)	88 (66)	229 (82)	
3+ visits	95 (23)	46 (34)	49 (18)	<0.001

^a^ Denotes statistical significance. p <0.05 considered statistically significant

^b^ Defined as one or more housing status changes within a 1 year follow-up period from index ambulatory visit

^c^ Health care utilization data collected over a 1 year follow-up time period from index ambulatory visit

### Effects of Housing Status on Frequent Emergency Room Use

**Table 2**shows the multivariable analysis stratified by housing status with frequent ER use as the outcome variable. In this multivariable analysis, there were no significant predictors of frequent ER visits in the housed patients. When adjusting for age, sex, race, housing status and stability, health characteristics, health care utilization, and mental health disorder among the homeless patients in the multivariable analysis, hepatitis C was the only significant predictor of frequent ER visits (OR 4.49, p<0.01). HIV seropositivity was not a significant predictor of frequent ER visits in either housed (adjusted OR 0.62, p = 0.30) or homeless (adjusted OR 0.0.44, p = 0.08) patients (**Table 2)**. In the pooled multivariable analysis using both unclustered and clustered data, there were no significant differences between the results (**Table 3**). Hepatitis C was the only significant predictor of ER visits in the unclustered (adjusted OR 2.84, p<0.001) and clustered (adjusted OR 2.49, p<0.001) data analyses.

**Table 2:** Multivariable Analysis: Predictors of Frequent Emergency Room Visits by Housing Status, Using Clustered Data.

	Housed patients (n = 134)		Homeless Patients (n = 278)	
Variable	Adjusted OR(Standard error)	p value	Adjusted OR(Standard error)	p value
**Demographics**				
**Age in years**				
18–54	Reference		Reference	
> 55	3.06 (1.90)	0.07	1.31 (0.62)	0.57
**Sex**				
Male	Reference		Reference	
Female	0.99 (0.96)	0.99	1.08 (0.52)	0.87
**Race**				
White	Reference		Reference	
Minority/Unreported	2.54 (1.94)	0.22	1.08 (0.49)	0.86
**Housing changes** ^**b**^				
No housing changes	Reference		Reference	
1+ housing changes	4.52 (3.98)	0.09	0.52 (0.34)	0.31
**Health Characteristics** ^**c**^				
Illicit drug use	1.87 (1.62)	0.47	2.53 (1.83)	0.20
Alcohol Use	0.50 (0.48	0.47	1.57 (0.94)	0.45
Tobacco use	1.26 (0.64)	0.65	0.55 (0.21)	0.12
HIV seropositive	0.62 (0.29)	0.30	0.44 (0.21)	0.08
Mental Health Disorder	2.35 (2.40)	0.40	1.67 (0.71)	0.23
Hepatitis C ^a^	0.93 (0.46	0.89	4.49 (1.88)	<0.01
**Charlson index score(excluding HIV)**				
0–1	Reference		Reference	
> 1	0.84 (0.20)	0.47	1.17 (0.28)	0.50
**Albumin**				
<3.5 g/dL	1.10 (0.79)	0.89	0.58 (0.51)	0.54
> 3.5 g/dL	Reference		Reference	
Missing	0.08 (0.08)	0.01	0.83 (0.38)	0.69
**Utilization** ^d^				
3+ Primary care visits	0.85 (1.52)	0.93	2.57 (1.53)	0.11
3+ Behavioral health visits ^e^	—-	—-	1.35 (1.06)	0.71
3+ Respite stays	3.89 (2.61)	0.04	0.96 (0.53)	0.94
+ Case management visits	0.71 (0.49)	0.62	1.53 (0.75)	0.39

^a^ Denotes statistical significance. p <0.05 considered statistically significant.

^b^ Defined as one or more housing status changes within a 1 year follow-up period from index ambulatory visit.

^c^ Reference group for these categories are patients without these health characteristics.

^d^ Health service utilization data collected over a 1 year follow-up time period from index ambulatory visit.

^e^ There were no behavioral health visits in homeless patients

**Table 3:** Pooled Multivariable Analyses: Predictors of Frequent ER Visits Using Unclustered and Clustered Data.

Variable	Unclustered data		Clustered data	
Demographics	Odds Ratio (Standard Error)	p value	Odds Ratio (Standard Error)	p value
**Age in years**				
18–54	Reference		Reference	
> 55	1.60 (0.52)	0.15	1.55 (0.60)	0.96
**Sex**				
Male	Reference		Reference	
Female	1.07 (0.39)	0.86	0.98 (0.40)	0.69
**Race**				
White	Reference		Reference	
Minority/Unreported	1.31 (0.47)	0.44	1.16 (0.43)	0.29
**Housing status**				
Housed	Reference		Reference	
Homeless	1.61 (0.58)	0.18	1.52 (0.61)	0.25
**Housing changes** ^**b**^				
No housing changes	Reference		Reference	
1+ housing changes	0.73 (0.35)	0.50	0.81 (0.39)	0.66
**Health Characteristics** ^**c**^				
Illicit drug use	1.71 (0.78)	0.24	2.05 (1.01)	0.14
Alcohol Use	1.98 (0.98)	0.17	1.37 (0.72)	0.55
Tobacco use	0.83 (0.27)	0.56	0.78 (0.23)	0.39
HIV seropositive	0.60 (0.22)	0.16	0.54 (0.18)	0.07
Mental Health Disorder	2.07 (0.79)	0.06	1.72 (0.66)	0.16
Hepatitis C ^a^	2.84 (0.98)	<0.001	2.49 (0.80)	<0.001
**Charlson index score(excluding HIV)**				
0–1	Reference		Reference	
> 1	1.02 (0.19)	0.91	0.99 (0.19)	0.98
**Albumin**				
<3.5 g/dL	0.97 (0.61)	0.96	1.09 (0.55)	0.87
> 3.5 g/dL	Reference		Reference	
Missing	0.72 (0.26)	0.35	0.60 (0.22)	0.15
**Utilization** ^d^				
3+ Primary care visits	2.17 (1.15)	0.15	2.11 (1.08)	0.15
3+ Behavioral health visits	0.90 (0.68)	0.88	0.95 (0.61)	0.94
3+ Respite stays	1.10 (0.42)	0.79	1.13 (0.46)	0.76
3+ Case management visits	0.95 (0.38)	0.90	1.12 (0.39)	0.75

^a^ Denotes statistical significance. p <0.05 considered statistically significant.

^b^ Defined as one or more housing status changes within a 1 year follow-up period from index ambulatory visit.

^c^ Reference group for these categories are patients without these health characteristics.

^d^ Health service utilization data collected over a 1 year follow-up time period from index ambulatory visit. Reference group was 0–2 visits per year.

### Subgroup analysis of Patients with History of Illicit Drug Use

In the subgroup analysis of patients with histories of illicit drug use (n = 287), when adjusting for age, sex, race, housing status and stability, health characteristics, and health service utilization, mental health disorder (OR 2.53, 95% CI 1.07–5.95) and hepatitis C (OR 2.85, 95% CI 1.37–5.93) were the most significant predictors of frequent ER use. (**Table 4).** HIV seropositivity was significantly associated with lower odds (adjusted OR 0.45, 95% CI 0.21 – 0.97) of frequent ER visits. **(Table 4).** Patient demographics and health care utilization were not predictive of frequent ER visits in this population.

**Table 4:** Predictors of Frequent Emergency Room Visits in Patients with History of Illicit Drug Use.

	Patients with History of Illicit Drug use n = 287	
Variables	n (%)	Adjusted OR (95% CI)
**Demographics**		
**Age in years**		
18–44	202 (70)	Reference
> 55	85 (30)	1.85 (0.92–3.74)
**Sex**		
Male	215 (75)	Reference
Female	72 (25)	0.81 (0.37–1.80)
**Race**		
White	83 (29)	Reference
Minority/Unreported	204 (71)	1.28 (0.5–2.77)
**Housing status**		
Housed	92 (32)	Reference
Homeless	195 (68)	1.86 (0.85–4.07)
**Housing changes** ^**b**^		
No housing changes	247 (86)	Reference
1+ housing change	40 (14)	0.70 (0.26–1.90)
**Health Characteristics** ^c^		
Alcohol Use	246 (86)	1.27 (0.43–3.76)
Tobacco Use	189 (66)	0.86 (0.42–1.74)
HIV seropositive	118 (41)	0.45 (0.21–0.97)
Mental Health Disorder ^a^	205 (71)	2.53 (1.07–5.95)
Hepatitis C ^a^	124 (43)	2.85 (1.37–5.93)
Charlson Index > 1	23 (8)	1.08 (0.74–1.58)
**Utilization** ^d^		
3+ Medical visits	242 (84)	2.20 (0.68–7.14)
3+ Behavioral Health visits	14 (5)	1.24 (0.27–5.62)
3+ Respite stays	69 (24)	1.11 (0.49–2.49)
3+ Case management visits	81 (28)	0.94 (0.41–2.17)

^a^ Denotes statistical significance. p <0.05 considered statistically significant.

^b^ Defined as one or more housing status changes within a 1 year follow-up period from index ambulatory visit.

^c^ Reference group for these categories are patients without these health characteristics.

^d^ Health service utilization data collected over a 1 year follow-up time period from index ambulatory visit. Reference group was 0–2 visits per year.

## Discussion

In this cohort of patients enrolled in a health care program for homeless persons, health characteristics, health service utilization, and predictors of frequent ER visits differed by housing status. In the descriptive analysis, housed patients were more likely to be older, have a history of alcohol use, mental health disorders, higher albumin levels, and had more primary care and case management visits. Our finding that housed patients were older and had more comorbidities, as well as significantly more histories of alcohol use and mental health disorders than homeless patients, may indicate that more housing opportunities are available to individuals with significant health problems and/or simply that engagement in case management services may be the precipitant to obtaining housing.

In the overall analysis, the only significant predictor of frequent ER visits in homeless patients was hepatitis C. These results suggest that hepatitis C may serve as a marker to identify patients at risk for frequent ER use. Prior literature has shown hepatitis C infection is associated with medical complications from unsafe drug and needle sharing behaviors, risky sexual behaviors and drug-related overdose ER visits [25–27]. Ascertaining whether or not the severity of hepatitis C infection is driving ER utilization is beyond the scope of this study, however we do surmise that hepatitis C is likely a marker for risks mentioned above. Interestingly, in this study hepatitis C was a significant predictor of ER use in homeless, but not housed individuals. Prior research has identified homelessness as a risk factor for hepatitis C [28], thus suggesting that stable housing could play a role in the prevention of hepatitis C and its associated risks. These results may also have implications for improving treatment for hepatitis C patients. If primary care programs for homeless individuals focus on hepatitis C management and treatment, the expense of newer, potentially curative, hepatitis C pharmacotherapeutics may be mitigated by a decrease in ER visits. Given that hepatitis C is a risk factor for frequent ER visits, one might consider the development of care models that allow for the ER to facilitate hepatitis C treatment follow up in the primary care setting.

In the subgroup analysis of patients with history of illicit drug use, hepatitis C and mental health disorders were the significant predictors of frequent ER visits. Interestingly, HIV serostatus did not play a significant role in frequent ER visits, likely because HIV seropositive patients enrolled in the health care program tend to be engaged in comprehensive outpatient care [29]. Our findings regarding the relationship between mental health disorders and ER use are consistent with findings from earlier studies where increased ER utilization is associated with mental health disorders [30–34]. Prior literature has shown that developing integrated care programs that identify challenging patients with mental health disorders can reduce emergency visits and hospitalizations [35–36]. Mental health disorders, hepatitis, and homelessness have all been identified as risk factors for emergency room visits in the literature [9, 37–38]. The results of this subgroup analysis suggest that supportive housing, mental health services integrated with primary care and easily accessible to people without housing, and linkage to care for drug users with hepatitis C patients could help reduce the burden on emergency services. People who inject drugs frequently present to the ER, often for complications of drug use not amenable to outpatient management [39–41]. Buprenorphine maintenance treatment has been shown to decrease emergency room utilization; therefore, the ER might also play an important role in referring persons who inject drugs for medication assisted therapies [42]. Prior literature has shown that patients with a history of drug use do, in fact, access medication assisted therapies when made available [43].

There were some limitations of this study. The study population is unique, in that patients are enrolled in a health care program for people experiencing homelessness that delivers comprehensive ambulatory care services, but the use of this population raises issues of generalizability. However, studying a population who generally has access to ambulatory health care services is suited to addressing the purpose of the study, which was to identify risks for ER in homeless patients despite access to ambulatory services. The study population was a convenience sample selected by HIV serostatus and matched on age, sex and housing status, which does also limit generalizability. While we felt that including the illicit drug use subgroup in the analysis was clinically relevant, due to the nature of the sampling, it is also important to recognize that this subgroup is not necessarily representative of unstably housed and homeless individuals who use illicit drugs. This study was also limited to one health care program and one urban medical center, and therefore did not account for ER visits that could have occurred elsewhere. Due to the study’s retrospective design, missing data were also an issue. We tried to address this issue by extracting data from progress notes and laboratory data instead of simply relying on diagnostic coding, however there were still some variables, such as length of homelessness and incarceration that could not be incorporated into the analysis due to missing data. In addition, we acknowledge that people experience episodic homelessness along a spectrum, which makes it challenging to confine individuals to one housing category. We attempted to address this issue by creating a variable to account for housing status changes. Despite these limitations, this study still sheds light on important risk factors for frequent ER visits in a homeless population. Future research involving patient perspectives on ER utilization use and barriers to linkage of care would enhance the findings from this study. Incorporating patient perspectives and our study's results into a tool such as a risk-based index to identify patients most at risk for frequent ER visits may ultimately help to identify patients requiring more targeted services.

## Conclusions

In a population enrolled in a health care for the homeless program that provides comprehensive outpatient services, hepatitis C was a strong predictor of frequent ER visits in homeless patients, but not housed patients. Specifically in a subgroup of patients with a history of illicit drug use, both hepatitis C and mental health disorders were strongly predictive of frequent ER visits. These findings suggest that in order to prevent frequent use of emergency services, providing stable, supportive housing, and targeting services towards homeless patients with mental health disorders and hepatitis C should remain public health priorities.
